# Metastable Austenitic Steel Structure and Mechanical Properties Evolution in the Process of Cold Radial Forging

**DOI:** 10.3390/ma12132058

**Published:** 2019-06-26

**Authors:** Dmitry Panov, Alexey Pertsev, Alexander Smirnov, Vladislav Khotinov, Yuri Simonov

**Affiliations:** 1 Department of Metal Science, Thermal and Laser Processing of Metals, Perm National Research Polytechnic University, 29 Komsomolsky prospekt, 614990 Perm, Russia; 2 Department Chief Metallurgist, Perm Scientific-Research Technological Institute, 41 Geroev Khasana Street, 614990 Perm, Russia; 3 Department of Materials Science in Engineering, Novosibirsk State Technical University, 20 Prospekt K. Marksa, 630073 Novosibirsk, Russia; 4 Department of Heat Treatment and Metal Physics, Ural Federal University, 19 Mira street, 620002 Ekaterinburg, Russia

**Keywords:** metastable austenitic stainless steel, cold radial forging, twinning, strain-induced martensitic transformation, band structure, strengthening, impact strength

## Abstract

The article presents the influence of structure formation on the properties of 321 metastable austenitic stainless steel in the process of cold radial forging (CRF). The steel under study after austenitization was subjected to CRF at room temperature with degrees of true strain (e) 0.26, 0.56, 1.00, 1.71 and 2.14. It has been shown that structure formation of the studied steel during CRF consists of three stages: formation of the lamellar structure of austenite, formation of the trapezoidal structure, and formation of the equiaxial grain structure. The kinetics of the strain-induced α′-martensitic transformation is related to the stages of structure evolution. Hardness, ultimate tensile strength and yield strength uniformly increase in all stages of structure formation with a significant decrease of elongation to fracture during the first stage of structure formation while the value of elongation to fracture remains constant in the subsequent stages of deformation. Impact strength of fatigue cracked specimens (KCT) decreases sharply at the first stage of structure formation and smoothly increases at the second and third stages. However, the impact strength of V-notch specimens (KCV) continuously decreases when deformation degree increases in the overall investigated deformation range.

## 1. Introduction

Metastable austenitic stainless steels (MASS), along with high corrosion resistance, impact strength and plasticity, have a rather low yield strength, which significantly limits the scope of their application. One of the most common ways to increase the yield strength of MASS is cold plastic deformation. It should be noted that significant structural changes occur as a part of this process. These changes are associated with the glide of dislocations, strain-induced twinning, formation of shear bands, precipitation or dissolution of particles, strain-induced martensitic transformation (SIMT), rotation of substructure elements and recrystallization [1,2,3]. Features of the mechanisms of structure formation during deformation of metastable austenite depend primarily on the temperature relationship of the stacking fault energy (SFE) and the crystallographic orientation of the crystals relative to the loading axis. According to the work [4,5], SIMT develops in the lower part of the temperature scale at deformation of metastable austenitic steels, and at lower temperatures ε-martensite is formed in greater quantities than at higher temperatures; further increase in temperature causes inhibition of SIMT and the intensification of twinning; at higher temperatures, deformation is accompanied by sliding dislocations and dynamic recrystallization. However, in the case of deformation with relatively low degrees at a constant temperature, when the development of SIMT and twinning is simultaneously possible, the mechanism of deformation in each grain is determined primarily by its orientation relative to the loading axis [6]. During deformation of MASS with different degrees, the process of structure formation can be divided into stages due to the implementation of various deformation mechanisms [3,7,8,9], which has a decisive influence on the mechanical properties of MASS.

At present, the processes of formation of structure and properties during cold plastic deformation of MASS by rolling [8,10,11], torsional deformation [12], surface mechanical attrition treatment [13] and equal-channel angular pressing [14,15,16] have been the most commonly studied processes. These methods of strain hardening differ in the strain pattern and rate, which, as is well known, significantly affect the processes of structure formation during deformation processing, and consequently the properties of the processed steel [3,13,17,18,19]. However, structural changes and their impact on the formation of a complex of mechanical properties of MASS during cold radial forging (CRF) are currently understudied.

It is known that during radial forging, the plastic flow develops inhomogeneously, and at the same time a fairly soft plastic deformation pattern is formed, which is close to uniform triaxial compression [20,21]. This allows us to form high degrees of non-destructive deformation, which is combined with a fairly high productivity of the processing. Thus, the paper is dedicated to the research of peculiarities of the formation of the structure and properties of MASS during CRF in order to study how the staging of the structure formation influence the behavior of mechanical properties.

## 2. Materials and Methods 

Within the present research, the commercial MASS was studied, which chemical composition is given in Table 1. It should be noted that the composition of the steel being studied corresponds to AISI 321 grade. 

The initial rod ø33 mm of the 321 MASS being studied was obtained by hot forging within the temperature range of 1220–900 °C with subsequent cooling in the open air to the ambient temperature. Preliminary thermal treatment consisted of heating to 1050 °C, holding within one hour and subsequent cooling in water. After such treatment, the steel was in an austenitic state with the average grain size of 10 ± 1 µm. In addition, the structure showed a certain amount of large irregular-shaped particles of primary titanium carbonitride and rod axis oriented fields of δ-ferrite, the content of which did not exceed 2%. Then, the rod of the steel observed was deformed at room temperature in a radial forging machine SXP-16 with four radially moving hammers in the following mode: workpiece feeding rate 180 mm/min, stroke frequency 1000 blows per minute, workpiece rotation speed 25 rpm. The deformation center was water-cooled during forging. Forging was performed in five steps: deformation from ø33 to ø29 mm, from ø29 to ø25 mm, from ø25 to ø20 mm, from ø20 to ø14 mm and from ø14 to ø11.3 mm, which made up 0.26, 0.56, 1.00, 1.71 and 2.14 of true strain, respectively. The true strain (e) was calculated using the following equation [22]:

(1) $$e=\ln\left(\frac{A_{0}}{A_{i}}\right),$$

where $A_{0}$ and $A_{i}$—rod cross-sectional area before and after deformation, respectively.

Transmission electron microscopy (TEM) was performed on thin foils with the use of the FEI Tecnai 20 G2 TWIN microscope (Novosibirsk State Technical University, Novosibirsk, Russia) at the accelerating voltage of 200 kV. Thin foils were obtained by means of preliminary cutting of flat templates with the thickness of 200 µm on aspark erosion machine from the central part of rods in cross and longitudinal axial section. Then, double-sided thinning was performed with the use of abrasive paper with P360, P800, P1200, P2000 grit size. Subsequent jet electropolishing of discs of 3 mm in diameter and thickness of 100 µm was performed with TenuPol-5 until perforation with electrolyte cooled down to −40 °C and consisting of 95% of acetic acid (CH_3_COOH) and 5% of chloric acid (HClO_4_).

Hardness was determined on microsections with the use of the Vickers hardness tester IT 5010, following the method of reconstructed indentation. To acquire microsections, samples were preliminarily press-fitted into the bakelite resin with the CitoPress-30 press. Then, a surface of the given samples was finished by the Tegramin machine in automatic mode with grinding wheels of different grit. Final polishing of the samples was performed with Al_2_O_3_ emulsion with 250 nm fraction. In the course of measurement, a tetrahedral diamond pyramid with square bottom was pressed into the surface of the microsection at the load of 50 g within 10 s, and then the mean value of the indentation’s diagonal lengths was determined. Hardness of the rod center and edge was determined as an arithmetic mean of five measurements. Hardness of the rod center was measured directly in the central part of the workpiece, and the hardness of the rod edge was measured in the area 2 mm away from the workpiece edge. The study of distribution of hardness over the cross section of the rod was performed in two mutually perpendicular directions with subsequent averaging of results of these two measurements for one sample.

Tensile tests were conducted by the Instron 5882 electromechanical testing system (Perm National Research Polytechnic, Perm, Russia) at room temperature at the strain rate of 1 × 10^−3^ s^−1^. Cylindrical specimens were cut from the central part of the rod in the axial direction with the diameter of the working section of 5 mm and the basic length of 25 mm. Following the tensile testing results, ultimate tensile strength (σ_B_), yield strength (σ_0.2_), elongation to fracture (δ) and area reduction (ψ) were determined.

Impact strength testing for evaluation of the impact strength were performed on the Instron 9350 (Ural Federal University, Ekaterinburg, Russia) test bench with the dropped weight (hammer displacement speed at the moment of impact −4.3 m/s) and in the KM-30 impact testing machine (hammer displacement speed at the moment of impact 5.5 m/s) at room and reduced temperatures (−20, −60, −160, −190 °C) on V-notch specimens (KCV) with the dimensions of 5 mm × 10 mm × 55 mm and on precracked specimens (KCT) with the dimensions of 5 mm × 11 mm × 55 mm. Specimens were cut from the central part of the rod in the axial direction. Before growing the fatigue crack on the specimen, a V-shaped concentrator with a depth of 1.5 mm was applied, from the top of it a fatigue crack with a length of 1.5 mm was further grown. The number of cycles for growing the fatigue crack was more 3000. Cooling of specimens with liquid nitrogen was performed in the cryogenic chamber in the ethanol (*t*_test_ = −20, −60 °C) or in the air (*t*_test_ = −160, −190 °C). Temperature control was performed with the use of the TM-8N thermometer and a L-type thermocouple with accuracy 1 °C. After testing precracked specimens (KCT) in the central part of fractures near the mouth of the fatigue crack, microfractographic analysis was performed with the use of the Hitachi S-3400N Christmas Edition scanning electron microscope (SEM, Perm State University, Russia, Perm) at the accelerating voltage of 20 kV. With the use of the obtained images of the fracture microsurface, the size of ductile fracture dimples was determined in two mutually perpendicular directions with the use of the Olympus Stream Motion software 1.8 (Perm National Research Polytechnic, Perm, Russia).

To determine the content of the magnetic α-phase, a multifunctional eddy-current tester MVP-2M with the converter detector F010 was used. Measurements were made on microsections in longitudinal and cross sections about the rod axis in the center of the section. The mean value of ten measurements for each sample adjusted with the correction factor of 1.7 [23] was taken as the value of the α-phase content.

## 3. Results

### 3.1. Structural Study

**Figure 1:**
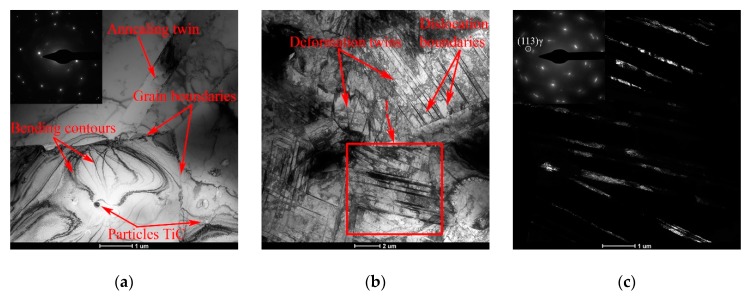

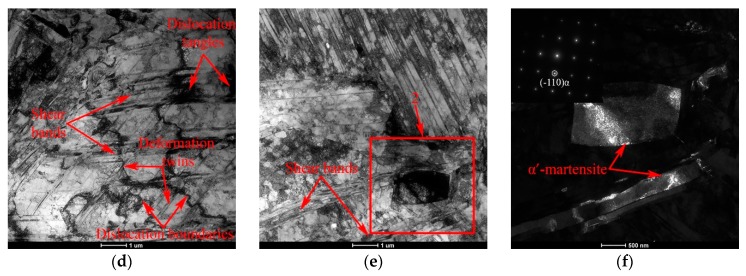
The TEM micrographs of the 321 MASS in the cross section of the rod in the initial state (**a**), after deformation with degrees of *e* = 0.26 (**b**,**c**) and *e* = 0.56 (**d**–**f**): (**a**,**b**,**d**,**e**) Bright-field images; (**c**,**f**) Dark-field images from sections 1 and 2 in the reflections (131)_γ_ and (−110)_α_, respectively.

In the initial structure of the rod, a predominantly austenitic grain structure is observed. Wide annealing twins are located within the austenitic grain, that is, they are limited by its boundaries; in some cases, at the place of contact of the annealing twin with the neighboring grain, bend contours are observed as a result of an increased level of structural stresses (Figure 1a). Inside austenitic grains, there are also randomly distributed single dislocations and dispersion particles of titanium carbide, which are mainly located on dislocations.

As a result of CRF with the degree of *e* = 0.26, a cellular dislocation structure is formed in the cross section of the workpiece studied. This structure is additionally fragmented by deformation twins as a result of the development of twinning-induced plasticity (TWIP effect) (Figure 1b,c). The deformation twins intersect the previously formed dislocation boundaries of the deformation cells, and then the process of the formation of a cellular dislocation substructure proceeds independently in volumes bounded by twins. 

**Figure 2:**
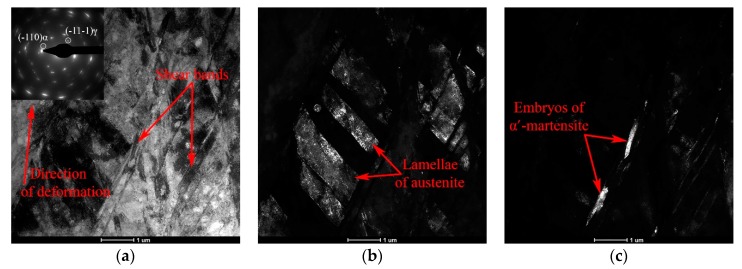
The TEM micrographs of the 321 MASS in the longitudinal section of the rod after deformation with the degree of *e* = 0.56: (**a**) Bright-field image; (**b**,**c**) Dark-field images from image (**a**) in reflections (–11–1)_γ_ and (−110)_α_, respectively.

Increasing the degree of deformation up to *e* = 0.56 leads to an increase in the twinning intensity inside austenitic grains. In other words, the proportion of microvolumes in which twinning develops in two systems increases, shear bands additionally appear (Figure 1d). Finally, it results in a predominantly lamellar substructure in the cross section of the rod (Figure 1e). The substructure is partially composed from trapezoidal blocks bounded by deformation twins (Figure 1d). In addition, after deformation with a degree of *e* = 0.56, embryos of deformation-induced α′-martensite were detected in the structure. These embryos additionally fragment the transverse structure of steel and are a sign of the developing transformation-induced plasticity (TRIP) effect (Figure 1e,f). In the longitudinal section of the rod, the detected shear bands are oriented parallel or at an angle of 25–30° to the deformation axis (Figure 2a). These shear bands form austenite areas with a lamellar structure along the axis of the rod (Figure 2b). The embryos of α′-martensite are located predominantly on fine deformation twins and inside the shear bands (Figure 2c).

**Figure 3:**
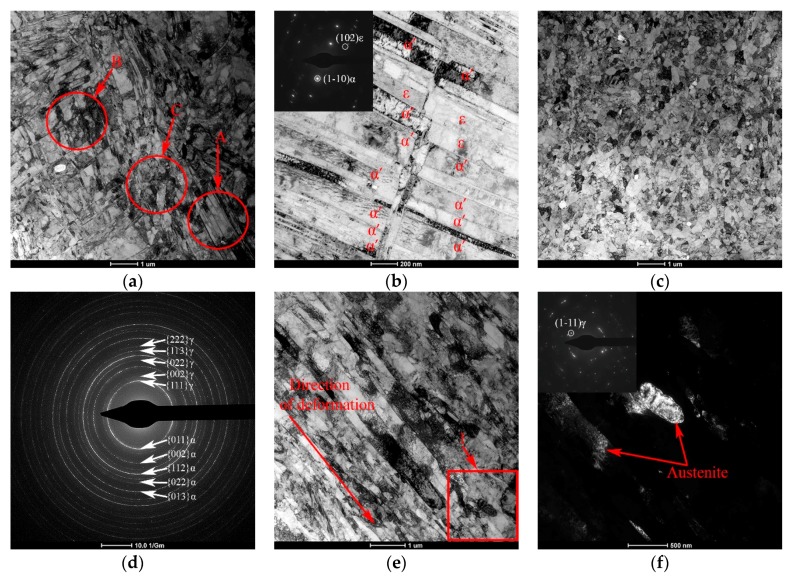
The TEM micrographs of the 321 MASS after deformation with the degrees of *e* = 1.00 (**a**,**b**) and *e* = 2.14 (**c**–**f**) in the cross (**a**–**d**) and longitudinal (**e**,**f**) sections of the rod: (**a**–**c**,**e**) Bright-field images; (**d**) Microdiffraction image from image (**c**); (**f**) Dark-field image of section 1 from image (**e**) in the reflection (1–11)_γ_.

As a result of the increased deformation degree up to *e* = 1.00 and *e* = 1.71, the structure is transformed from predominantly lamellar to block trapezoidal and further to equiaxed grain, as evidenced by the presence of three types of areas in the structure (Figure 3a):

- Microvolumes of austenite and martensite with a lamellar substructure (indicated by letter A in Figure 3a). However, the number of such areas is relatively small after deformation with *e* = 1.00, and almost equal to zero after deformation with *e* = 1.71;
- Microvolumes with block trapezoidal substructure, formed as a result of twinning of austenite in two systems (indicated by letter B in Figure 3a);
- Microvolumes where the transformation of the block trapezoidal substructure into an equiaxed grain structure occurs (indicated by letter C in Figure 3a).

Areas of lath martensite consisting mainly of α′-martensite plates with a body-centered cubic (BCC) crystal lattice are also observed in the structure, however, according to the dark-field analysis, a small amount of ε-martensite with a hexagonal close-packed (HCP) crystal lattice is present in the package structure (Figure 3b). 

CRF with the degree of *e* = 2.14 leads to the formation of an equiaxed grain structure in the cross section of the rod (Figure 3c). The deformation twins are virtually undetected inside the formed elements of the structure of an equiaxed shape. The microdiffraction pattern here consists of closed rings due to the large number of micro-volumes of austenite and martensite present with different crystallographic orientations (Figure 3d). In the longitudinal section of the rod, a two-phase martensitic-austenitic band structure oriented along the deformation axis is observed (Figure 3e,f), the formation of which is caused by the gradual accumulation of shear bands and the formation of deformation-induced α′-martensite within these bands. This band structure is fragmented in the transverse direction by dislocation and interphase boundaries.

**Figure 4:**
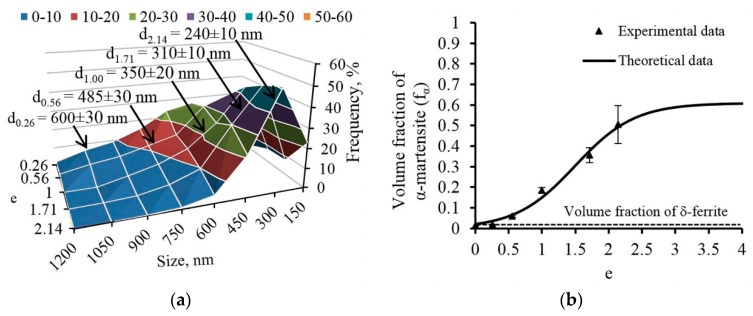
(**a**) The size distribution of substructure elements of the 321 MASS after deformation with different degrees; (**b**) The proportion of deformation-induced α′-martensite in the structure according to the experiment (points) and the approximation of experimental data (line) depending on true strain. Note: *d_e_* is the average size of the substructure elements, which were obtained after deformation with the degree *e*.

The full quantitative evaluation of the dislocation and twins density, as well as the size of the substructure elements was carried out in the previous work [24]. The TEM micrographs were used to estimate the size of the substructure elements of the steel being studied, by which the size of relatively dislocation-free regions bounded by dislocation, twinning, intergranular or interphase boundaries was meant. The results are presented by a surface formed by lines representing the size distribution of these elements after various degrees of deformation (Figure 4). Thus, a gradual decrease in the average size of a substructure element with an increase in the degree of deformation from 600 ± 30 nm after *e* = 0.26 to 240 ± 10 nm after *e* = 2.14 is shown. 

Figure 4b shows a dependence of the content of deformation-induced α′-martensite in the studied 321 MASS on the degree of deformation considering the contribution of δ-ferrite (1.5%). The content of deformation-induced α′-martensite is close to a zero level after the deformation *e* = 0.26. However, a noticeable increase in the amount of the α-phase due to the formation of deformation-induced α′-martensite is observed after *e* = 0.56. Further deformation leads to a significant acceleration of the γ → α′ transformation due to an increasing number of areas of predominant formation of α′-martensite embryos—deformation twins, shear bands and grain boundaries [3]. Furthermore, Figure 4b shows an approximating sigmoidal curve, which will be discussed in Section 4.

### 3.2. Hardness Testing

**Figure 5:**
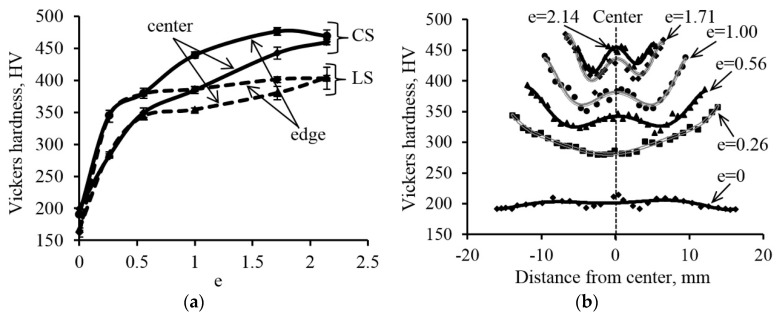
The results of the Vickers hardness study of the 321 MASS after various degrees of deformation: (**a**) The dependence of the center and the edge hardness in the cross section (CS–cross section) and the longitudinal section of the rod (LS—longitudinal section); (**b**) The hardness distribution over the cross section of the rod after deformation with different degrees.

Figure 5a shows that the hardness in the center and on the edge of the rod in the cross and longitudinal sections is almost the same in the initial state. As the cold plastic deformation increases, so does the overall hardness level, yielding a difference in the hardness of the center and the edge of the rod. The overall level of steel hardness in the cross and longitudinal sections is the same up to deformation with the degree of *e* = 0.56. The hardness level is greater in the cross section than in the longitudinal one for large degrees of deformation, and the difference of hardness between the center and the edge of the rod decreases, reaching the same level after deformation with the degree of *e* = 2.14. This phenomenon is associated with strain-induced martensitic transformation in the process of radial forging, when a significant amount of martensitic is formed at degrees of strain above *e* = 0.56. Apparently, strain-induced martensite is formed with a certain crystallographic orientation relative to the loading axis [3,6], which might lead to different hardness in different sections. However, this phenomenon requires a separate and additional study needs further research. 

A detailed study of the hardness distribution in the cross section of the rod found that the hardness of the steel is almost at the same level in the initial state (Figure 5b). After deformation with the degree of *e* = 0.26, a hardness gradient appears over the cross section from the center to the edge. Increasing degrees of deformation to *e* = 0.56, 1.00 and 1.72 causes a further increase in the overall level of hardness and the appearance of a hardness peak in the core. With a subsequent increase in the deformation degree to *e* = 2.14, the hardness value of the core reaches the hardness level of the rod edge, but at the same time a minimum of hardness is observed at half the radius. Thus, a non-uniform hardness distribution is formed in the cross section of the rod in the process of cold radial forging.

### 3.3. Tensile Testing

**Figure 6:**
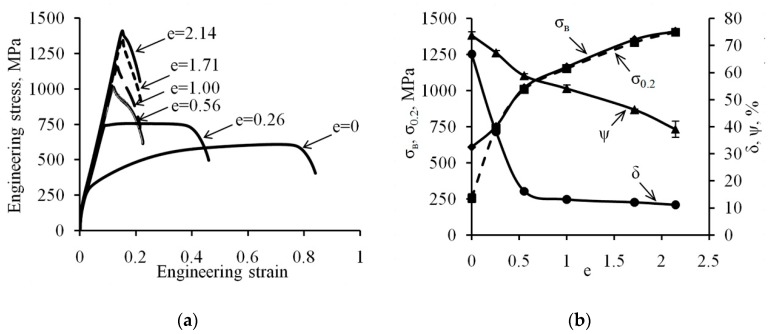
Results of tensile testing of the 321 MASS: (**a**) Stress-strain curves; (**b**) Relation of mechanical properties to the true strain value.

Figure 6a represents stress-strain curves for the steel under study. The considerate uniform elongation of the steel from the initial state of 55% to 67% can be observed, while the localized deformation only reached 12%. After deformation with the degree of *e* = 0.26, the stress-strain curve shows a long range of the specimen elongation development with no increase in the stress. In addition, the elongation to fracture is 26%, and the contribution of the localized deformation remains 12%. A further increase in the deformation degree within the range of *e* = 0.56 to *e* = 2.14 leads to a qualitative change in the diagram shape, actually implying a complete absence of the strain hardening section; while elongation to fracture for the specimens during the testing was mainly implemented due to the plastic deformation localization.

Relations of ultimate tensile strength (σ_B_) and yield strength (σ_0.2_) to the degree of the true strain are given in Figure 6b. In the initial state, the ultimate tensile strength and the yield strength are at the level of 610 and 260 MPa, respectively. An increased degree of cold plastic deformation induces an increase in ultimate tensile strength and yield strength, however, in the deformed state, these parameters practically coincide, which is due to almost complete lack of the strain-hardening effect. After deformation with the greatest degree of *e* = 2.14, the ultimate tensile strength made 1410 MPa with the yield strength of 1405 MPa. The value of the elongation to fracture (δ) for the steel under study decreases from 67% to 12% in the result of the deformation with the degree up to *e* = 0.56 and is practically maintained at this level with the increase in the deformation degree up to *e* = 2.14 (Figure 6b). The reduction of area (ψ) uniformly decreases from 74% to 39% within the whole range of deformation under study as the cumulative plastic deformation degree increases.

### 3.4. Impact Strength Testing

**Figure 7:**
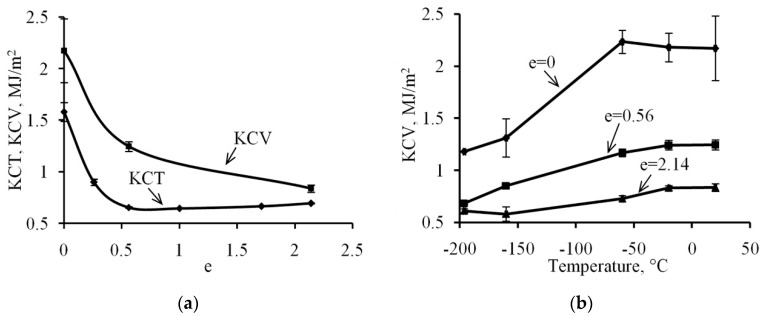
Results of impact strength testing of the 321 MASS after various degrees of deformation: (**a**) Relation of impact strength at room temperature to the true strain; (**b**) The temperature dependence of impact strength.

**Figure 8:**
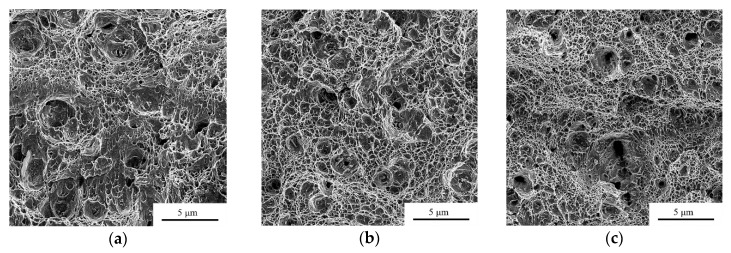

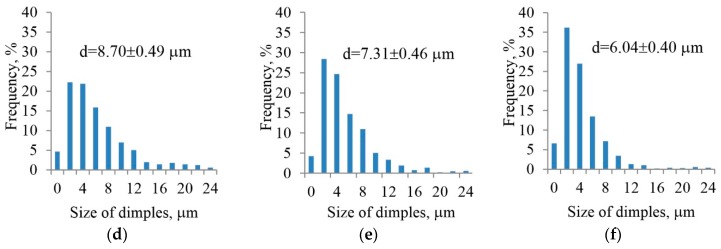
(**a**–**c**) SEM micrographs the fracture surface after the impact strength testing on KCT 321 MASS for (**a**) the initial state; (**b**) after deformation with degrees of *e* = 0.56; and (**c**) *e* = 2.14. (**d**–**f**) Distribution histograms of the size of the ductile fracture dimples of the fracture surface after the impact strength testing on KCT 321 MASS for (**d**) the initial state; (**e**) after deformation with degrees of *e* = 0.56; and (**f**) *e* = 2.14. Note: *d* is the average size of the ductile fracture dimples.

The impact strength of the V-notch specimens (KCV) at room temperature continuously decreases as the deformation degree increases from *e* = 0 to *e* = 2.14 (Figure 7a). However, the impact strength of the fatigue cracked specimens (KCT) drops as the deformation degree increases up to *e* = 0.56 from 1.58 MJ/m^2^ to 0.65 MJ/m^2^, while in the case of larger degrees of deformations a gradual KCT increase up to the level of 0.70 MJ/m^2^ is observed. The analysis of the fracture surface for the steel under study after completion of the impact strength tests of KCT at room temperature revealed that in all cases, dimples are the primary component of the fracture and the fracture occurs through the growth and coalescence of dimples (Figure 8). However, a higher level of impact strength in the original state shall be accompanied by a more developed micro-surface of fractures (Figure 8a). In the case of testing the specimens deformed with degrees of *e* = 0.56 and *e* = 2.14, the fracture microsurface is qualitatively similar and consists mainly of dimples (Figure 8b,c), which was confirmed by close values of KCT: 0.65 and 0.70 MJ/m^2^, respectively. The quantitative analysis of the fracture surface showed that with the structure refinement (Figure 4) by increasing the deformation degree causes the formation of a more dispersed fracture microsurface (Figure 8d–f). It should be noted that the share of large dimples with a diameter of 10 µm or more in steel fracture in its original state amounts to 25%, which exceeds the share of large dimples in the deformed specimens with degrees of *e* = 0.56 and *e* = 2.14, where the share of the large dimplesis 17% and 10%, respectively.

The relation of impact strength of steel V-notch (KCV) specimens made of steel under study in the initial state has an expressed thermal sensitivity within the temperatures range under study and decreases from 2.2 MJ/m^2^ at room temperature up to 1.2 MJ/m^2^ at the temperature of −190 °C (Figure 7b). The overall impact strength level of specimens made of steel deformed with a degree of *e* = 0.56 is lower compared to the initial state, while the thermal sensitivity remains as expressed as it was: the KCV impact strength reduces from 1.2 MJ/m^2^ at room temperature up to 0.6 MJ/m^2^ at the temperature of −190 °C. The lowest thermal sensitivity of the KCV impact strength is observed in the case of 321 MASS after deformation with the degree of *e* = 2.14 when KCV decreases from 0.8 to 0.6 MJ/m^2^ in the range from room temperature to −190 °C.

## 4. Discussion

### 4.1. Evolution of the 321 MASS Structure during CRF

#### 4.1.1. Stages in the Structure Formation Process

To identify the patterns of evolution in the structure of the 321 MASS, research was completed into the structural state in the longitudinal and cross section of a rod after varying degrees of deformation. According to the data given in reference [25], if the stacking fault energy level of the package (SFE) exceeds 35 mJ/m^2^, the deformation develops mostly by the gliding of dislocations; if SFE is within the range of 18 to 35 mJ/m^2^, then the deformation is accompanied by twinning; if SFE does not exceed 18 mJ/m^2^, then the formation of strain-induced martensite is mainly observed. However, when SFE is below 13 mJ/m^2^, then ε-martensiteis formed instead of α′-martensite [2]. According to different sources data, the 321 MASS under study has the SFE at the level of 13.2–20.9 mJ/m^2^ [26,27,28], indicating the borderline position of this steel, i.e., simultaneous inclination towards TWIP and TRIP effects.

**Figure 9:**
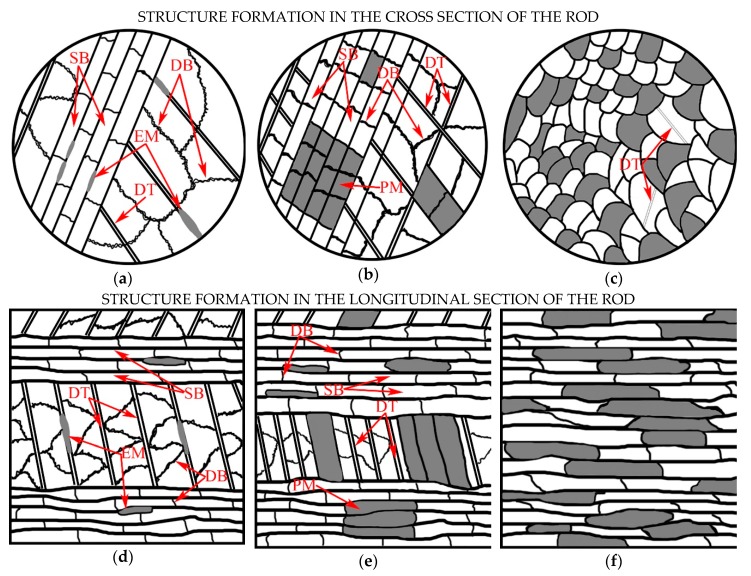
Scheme of the 321 MASS structure formation of the rod: (**a**,**d**) First stage; (**b**,**e**) Second stage; (**c**,**f**) Third stage. Note: white color: austenite; grey color: martensite; DB: dislocation boundary; DT: deformation twin; SB: shear band; EM: embryo of martensite; PM: packages of martensite.

Based on the results of the 321 MASS structure studies after varying degrees of CRF, three stages of structure formation can be clearly identified depending on the interval of realized degrees of deformation, which is presented in Figure 9. The first stage of the structure formation (Figure 9a) is characterized by deformation (with degrees up to *e* = 0.56) according to the twinning mechanism—TWIP effect, that forms the barrier for the glide of dislocations with the formation of dislocation cells (Figure 1b). The deformation twins cross the borderlines of previously formed dislocation cells and further act as barriers for the dislocations movement, which causes formation of dislocation agglomerates and cells inside the micro-volumes limited with deformation twins. Inside of austenitic grains, the twinning occurs mainly in one system, however, the twinning in two systems can be observed simultaneously in some micro-volumes. The deformation twins in steels with face-centered cubic lattice (FCC-lattice) are formed as a result of emission of the Shockley partial dislocation on the planes system {111} [29]. In addition to deformation twins, the structure includes the formed shear bands that split austenitic grains into micro-volumes of austenite with lamellar structure oriented in a predictable manner along the axis of the rod (Figure 9d). In addition, at such degrees of deformation, the embryos of strain-induced α′-martensite are formed in the structure. Thus, at the first stage of deformation, the lamellar austenitic structure is formed with the deformation twins and shear bands.

At the second stage of structure formation (deformation degree within the interval from *e* = 1.00 to *e* = 1.71), the process of strain-induced twinning on secondary systems is intensified, which leads to the transformation of the lamellar structure obtained at the first stage into a block trapezoidal structure in the cross section. However, it provides the ground for strain-induced martensite transformation (SIMT) in the result of the embryos formation and growth with further generation of martensite packages [30]. It should be noted that the twinning and SIMT development provides the ground for the formation of dislocation clusters and walls inside the substructure elements that are limited by deformation twins, which is consistent with the data contained in reference [5]. The latter paper traces the presence of the deformation dislocation mechanism within a wide temperature range in conjunction with other mechanisms of twinning. Thus, at this stage a block trapezoidal austenitic-martensitic structure is formed predominantly (Figure 9b) in the transverse direction, coupled with getting a band structure in the longitudinal section (Figure 9e).

At the third stage, in the majority of the studied microstructure fields of the rod cross section, the block trapezoidal structure is transformed into the equiaxed grain one (Figure 9c), which is probably caused by the rotation of each piece before gaining the equilibrium orientation [31]. In addition, the deformation twins are not detected in the majority of grains, except for the most coarse grains, which is due to the so-called restriction effect [3,32]. In longitudinal section at this stage, the band austenitic-martensitic structure is formed (Figure 9f), which is additionally fragmented with dislocation and interphase borderlines in the transverse direction.

The shear bands are not thinned during the deformation and only increase in their number. In other words, the average width of the lamella in the process of implemented deformation degrees is at the same level and corresponds to the diameter of the grain obtained at the deformation degree of *e* = 2.14 in cross section of a rod of 240 ± 10 nm [24]. It should be noted that the formation of a band structure in metastable austenitic steels in the deformation direction is recorded by other deformation methods [3,33], however, in our study the globular structure in the cross section indicates the columnar form of the formed grains.

#### 4.1.2. Restriction Effect of the Twinning Process

The restriction effect of deformation twins formation caused by the 321 MASS structure refinement was discussed in references [3,32]. Structure refinement reduces the propensity to twinning due to changes in the ratio of stresses related to complete dislocation formation or Shockley partial dislocation, which can be formulated as the expression to define the critical diameter of the FCC-lattice metal grain, in which one strain mechanism is switched with the other [34]:

(2) $$d_{c}=\frac{2\alpha \mu \left(b-b_{1}\right)b_{1}}{\gamma },$$

where *μ*—shear modulus that amounts to 77.4 GPa [2]; *b* and *b*_1_—strength of dislocation for complete dislocation and Shockley partial dislocation, which in this case amount to 2.54·10^−10^ m and $\frac{\sqrt{3}}{3}\cdot b$, respectively [32]; *α*—Taylor constant taken as equal to 1 [31]; γ—stacking fault energy, which at difference estimates is at the level of 13.2–20.9 mJ/m^2^ [26,27,28].

For the case here, the solution of this equation states that in theory the process of twinning is possible only for the states with a grain size exceeding 190 nm. This is satisfactorily consistent with the experimental results for the case after the deformation with the degree of *e* = 2.14 when the structure is formed with the characteristic element close to *d_c_*—240 ± 10 nm, where almost no twining is observed. However, there are works [9,14], which show twinning of nano-grains with sizes less than 100 nm in steels deformation of a similar chemical composition, which requires specific consideration.

Another possible reason for the almost complete absence of twins in the 321 MASS deformed structure is rod heating in the process of cold radial forging because of multiple dynamic impact of striking edges on the treated steel during CRF. According to some estimates, in the case of the dynamic deformation of metastable austenitic steel, the MASS heating at the deformation can reach 200 °C [3], which along with structure refinement facilitates the transition from the temperature deformation field to the twinning mechanism in the deformation area on the glide of dislocations mechanism [5]. However, in order to minimize this phenomenon, the deformation center was water-cooled in the process of deformation to room temperature.

#### 4.1.3. Strain-Induced Martensite Formation

The literature contains various data on the mechanism of strain-induced α′-martensite formation in 304-type or 321-type MASS. Thus, [11] refers to observing the shear bands formations right before the phase transformation in the austenite. Inside the bands, α′-martensite is formed according to γ→ε→α′ mechanism. In other works [3,7,35], the authors observed the formation according to γ→α′-transformation mechanism without intermediate ε-martensite formation. The differences seem to be caused by diverse in the chemical composition and deformation temperature, different original state and deformation procedure. Thus, for instance, steel heating in the process of deformation results in changing the mechanism from γ→ε→α′ to γ→α′-transformation [5,30].

In this work, at the first stage of structure formation (deformation up to *e* = 0.56) the embryos of strain-induced α′-martensite (Figure 9a,d) are formed on the deformation twins and inside the shear bands, which is in compliance with the following works [8,35]. With a subsequent increase in the deformation degree at the second stage, SIMT develops in austenite with lamellar structure, and martensite packages are formed. However, within a single package, α′-martensite areas are mainly observed, while dark-field analysis employing methods of TEM revealed a small amount of ε-martensite crystals (Figure 3b,c). These observations can be explained by the ability of independent development for γ→α′ and γ→ε transformations exposed to deformation, which has been shown in the SIMT study in-situ [36]. In addition, the total structure dispersion leads to the increase in the number of strain-induced α′-martensite, and their number continuously increases due to the formation and growth of new embryos. At the third stage, the previously formed martensite packages are distorted and mixed with the areas of non-transformed austenite, which is caused by the rotation and mutual offset of microvolumes during deformation [25].

Kinetics of strain-induced α′-martensite formation during deformation has an expressed sigmoidal nature (Figure 4b). In order to approximate the experimental data by the least squares method and kinetics description, the model was chosen to contain the main SIMT parameters [37]:

(3) $$f_{\alpha^{\prime }}=\frac{f_{s}}{(1+\exp\left(-\beta \left(e-e_{m}\right)\right)}$$

where *f_α_*_′_—volume fraction of α′-martensite formed after accumulation of true strain (*e*) in the current moment; *f_s_*—maximum volume fraction of α′-martensite formed during the restriction of transformation under such deformation conditions; *e_m_*—degree of true strain at 50% of *f_s_*; *β*—parameter defining the speed of transformation.

In the results determining the parameters of the equation (1), it was found that saturation of SIMT happens after the deformation at the level of *e* = 3.5 with the transformation volume fraction of 0.608. Maximum speed of the transformation is observed during deformation *e* = 1.5, which indicates the highest SIMT speed at the second stage of structure formation and its restriction at the third stage. This is due to the increase in the number of areas of formation and growth of α′-martensite embryos during the deformation with their subsequent exhaustion in the process of transformation [3] and the stabilization of austenite relative to SIMT due to the total structure refinement [38].

### 4.2. Mechanical Properties Evolution during CRF

This section discusses the change of mechanical properties depending on the peculiarities of the implemented structure formation process during CRF.

#### 4.2.1. Changes of Hardness and Strength during CRF

At all stages of structure formation with CRF, the hardness and strength characteristics gradually increase as the deformation degree increases (Figure 5b and Figure 6b), which is due to the accumulation of twins and dislocations at the first stage of structure formation, the formation of strain-induced martensite and increased dislocation density at the second stage, as well as the formation of the dispersed structure and strain-induced martensite at the third stage [8]. According to reference [39], the SIMT hardness is close to the hardness of the austenite volumes surrounding it, but it leads to additional dispersion of the structure, as its boundaries are additional barriers to the movement of dislocations [40], while the total dispersion of the structure causes increased levels of yield strength and hardness in accordance with the Hall-Petch relationship [3,14,41,42].

The distribution of hardness over the cross section of the rod after CRF is non-uniform: thus, with the view to the increase in the general level of hardness, the maximum hardness is formed in the center of the rod, while the minimum hardness occurs at the half of the radius. This allows radial forging to be considered as a method of obtaining the non-uniform rod in its cross-section (quasi-composite material), along with well known methods of formation of non-heterogeneous gradient materials such as torsional deformation [12] and surface mechanical attrition treatment [13]. This fact has a positive effect on the resistance to the transverse crack impeding during the impact strength testing of the received CRF one-phase material [43].

#### 4.2.2. Change of Plasticity and Impact Strength Testing during CRF

The total structure refinement leads to the stabilization of austenite relative to γ→α′-transformation including due to the decreasing of SIMT interval by the temperature scale [38], which significantly reduces steel plasticity during tensile tests due to no TRIP effect being present during the tensile tests [44]. Thus, at the first stage of structure formation, there has been a significant drop in elongation to fraction from 67% in its original state to 12% after deformation with a degree of *e* = 0.56 (Figure 6b), which is due to the occurrence of shear bands in the austenite structure, a significant increase of the density of dislocations and deformation twins [24]. As a result, there is an increase in the number of boundaries impervious to dislocations, which significantly complicates their movement and redistribution and leads to a drop in the relative elongation [8]. An increase in the deformation degree up to *e* = 2.14 does not cause a change in the level of elongation to fracture. The relative reduction of area with the increasing degree of the deformation gradually reduces from 74% in its original state to 39% after deformation with the degree of *e* = 2.14. Therefore, the behavior of elongation to fracture under uniaxial tensile tests depending on the accumulated degree of the deformation in the CRF process allows us to clearly identify the first stage of the structure formation during deformation.

Impact strength of pre-cracked specimens (KCT) at the first stage of the structure formation and the elongation sharply decrease from the level of 1.58 MJ/m^2^ in its original state to 0.65 MJ/m^2^ in the state after deformation with a degree of *e* = 0.56, and at the second and third stages, there is a smooth increase to 0.70 MJ/m^2^ after deformation with a degree of *e* = 2.14. If the value of the KCT impact strength obtained when testing the specimen with a crack shows the relative work of the crack propagation, the test of the V-notch (KCV) specimen shows the relative work of the nucleation and growing of cracks. Thus, the increase in the degree of deformation is accompanied by the decrease in the origination of cracks, as the difference between the KCV and KCT. Within the interval of deformation from *e* = 0 to *e* = 0.56, the relative work of crack growth drastically reduces, which is caused by accumulation of the number of deformation twins [24]; while during the deformation in the interval from *e* = 0.56 to *e* = 2.14, the relative work of crack growth slightly increases and the relative work for crack initiation is sensibly reduced (see Figure 7a). This is caused by the formation of the band structure in the cross-section, which complicates the growth of cracks in the transverse direction due to the increasing in number of inter-grains and inter-phase borderlines on the way of the crack growth, which are local ductility barriers.

The temperature dependence of the impact strength of the KCV V-notch specimens most clearly appears in the initial state and after deformation with the degree of *e* = 0.56, while impact strength drops almost by half in the temperature range from room temperature to −190 °C. In case of impact strength testing steel, which is deformed with a degree of *e* = 2.14, the impact strength decreases only by 25% in the investigated temperature range.

**Figure 10:**
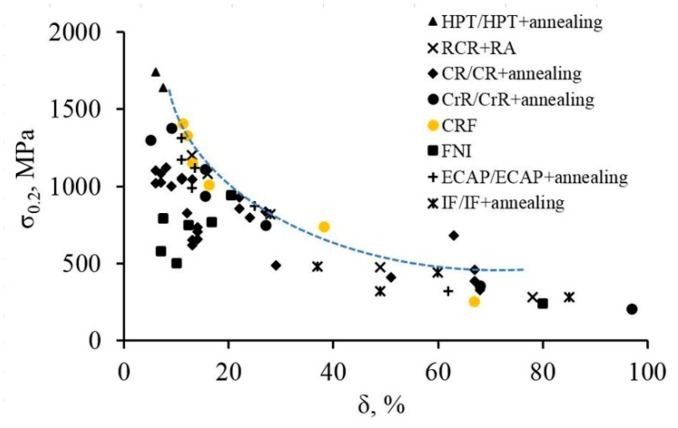
Yield strength—elongation to fracture relation of the 321 MASS after different strengthening treatment methods. Note: high pressure torsion (HPT) [45], repetitive cold rolling and reversion annealing (RCR + RA) [46], cold rolling (CR) [1,47], cryogenic rolling (CrR) [48,49], cold radial forging (CRF), fast neutron irradiation (FNI) [50], equal channel angular pressing (ECAP) [15,16], isothermal forging (IF) [49].

When compared with the results of mechanical properties tests on 321 MASS steel obtained by other authors after treatment using other methods of structure formation, such as cryogenic and repetitive cold rolling, equal-channel angular pressing, high-pressure torsion, fast neutron irradiation, as well as post-deformation annealing in some cases, we have shown that patterns of change in the yield strength and elongation to fracture coincide with the data presented by other authors (Figure 10). However, at the level of the yield strength of 740 MPa, the elongation to fracture value exceeds other treatment methods, and in a range of values above 1000 MPa, the results of this treatment are not inferior to the results of other treatment methods.

The research can be extended further to find the possibilities to increase the plasticity and strength without substantial reduction of strength properties through the process of recovery, recrystallization and reverse martensite transformation during post-deformation thermal treatment of the 321 MASS.

## 5. Conclusions

The results of the research of the 321 MASS microstructure evolution during CRF have shown that the structure formation during deformation with degrees up to *e* = 2.14 occurs in three stages in the rod cross section: in the first stage (up to *e* = 0.56), the lamellar structure of austenite is formed as a result of the formation of shear bands and deformation twins; in the second stage (*e* = 1.00–1.71), the lamellar structure is transformed into a trapezoidal one; in the third stage (*e* = 2.14), the equiaxed grain structure is formed. During CRF, the band austenitic-martensitic structure is formed in the longitudinal section. The kinetics of SIMT show that the onset is observed at the first stage when the martensite embryos formation occurs; the maximum speed of the transformation is registered at the second stage when α-martensite packages and a few areas of ε-martensite are formed; SIMT slows down at the third stage because of the exhausted areas of α′-martensite embryos formation and the stabilization of the remaining austenite due to the total structure refinement.

At all identified stages of structure formation during CRF, the hardness and strength characteristics almost uniformly increase with the overall structure dispersion. The distribution of hardness over the cross section of the rod after CRF is non-uniform. A significant drop of elongation to fracture is observed at the first stage of structure formation. It is caused by the fragmentation of austenite by deformation twins and shear bands. An increase in the deformation degree up to *e* = 2.14 does not cause a change in the level of elongation. Reduction of area smoothly decreases when the cumulative deformation degree increases. The impact strength of precracked specimens (KCT) as well as elongation decreases sharply at the first stage of structure formation, and there is a smooth increase at the second and third stages. However, the impact strength of V-notch specimens (KCV) continuously decreases when the deformation degree increases. The temperature dependence of the impact strength of the V-notch specimens (KCV) most clearly appears in the initial state and after deformation with a degree of *e* = 0.56, while the impact strength drops almost by half in the temperature range from room temperature to −190 °C. In case 321, MASS is deformed with a degree of *e* = 2.14, while the impact strength decreases only by 25% in the specified temperature range.

## Figures and Tables

**Table 1:** Content of chemical elements wt %.

Grade	C	Si	Mn	P	S	Cr	Ni	Ti	Fe
AISI 321	≤0.080	≤1.00	≤2.0	≤0.045	≤0.030	17.00–19.00	9.00–12.00	5 × C–0.70	basis
Studied steel	0.07	0.39	1.12	0.019	0.005	18.75	9.20	0.59	basis
